# Phytochemical Screening and *In Vitro* Antioxidant and Anticancer Evaluation of Stem and Leaf Extracts of *Cissampelos pareira* L.

**DOI:** 10.1155/mi/7555073

**Published:** 2025-07-11

**Authors:** Arthid Thim-uam, Chutamas Thepmalee, Wittaya Chaiwangyen, Napapan Kangwan, Ratchanaporn Chokchaisiri, Piyawan Nuntaboon, Angkana Songkrao, Amnart Onsa-ard

**Affiliations:** ^1^Division of Biochemistry, School of Medical Sciences, University of Phayao, Phayao 56000, Thailand; ^2^Division of Physiology, School of Medical Sciences, University of Phayao, Phayao 56000, Thailand; ^3^Division of Chemistry, School of Science, University of Phayao, Phayao 56000, Thailand

**Keywords:** anticancer activity, antioxidant activity, *Cissampelos pareira* L, phytochemicals, proximate composition analysis

## Abstract

This study was conducted to investigate the phytochemical constituents, antioxidant capacity, and anticancer potential of aqueous, ethanolic, and hexane extracts derived from the leaves and stems of *Cissampelos pareira* L. (*C. pareira* L.). Proximate analysis revealed that the stems contained a high fiber content (48.02% ± 0.14%) and carbohydrate content (32.31% ± 0.47%), whereas the leaves exhibited a higher carbohydrate content (53.93% ± 0.02%). Ethanolic extracts of both stems and leaves showed notably high levels of total phenolic and flavonoid compounds. Specifically, the total phenolic content (TPC) was 58.26 ± 2.56 mg GAE/g dry weight (DW) in the stem extract (CPSE) and 95.73 ± 1.76 mg GAE/g DW in the leaf extract (CPLE). The total flavonoid content (TFC) was 27.23 ± 2.89 mg CE/g DW in CPSE and 82.68 ± 4.98 mg CE/g DW in CPLE. Furthermore, the ethanol extract from *C*. *pareira* L. leaves exhibited the strongest free radical scavenging activity, with IC_50_ values of 55.00 ± 1.93 µg/mL and 25.77 ± 0.38 µg/mL against DPPH and ABTS radicals, respectively. In terms of anticancer activity, the hexane extract from the stem (CPSH) showed the highest inhibitory effects on the proliferation of breast cancer cell lines (MCF-7 and MDA-MB-231), as well as HEK293T cells. Interestingly, the proliferation of normal human dermal fibroblast (NHDF) cells was more effectively inhibited by CPLE. These findings indicate that the ethanol extract of *C*. *pareira* L. leaves possesses strong antioxidant properties, while the hexane extract of the stem demonstrates potent anticancer activity. Therefore, both leaf and stem extracts of *C*. *pareira* L. hold promise for development as functional foods or dietary supplements due to their diverse and significant biological activities.

## 1. Introduction

Free radicals are primarily generated through oxidative processes and are known to play significant roles in the deterioration of food quality and the degradation of various chemical compounds. In biological systems, they are implicated in the pathogenesis of numerous human diseases, including age-related disorders, cardiovascular diseases, cancer, and inflammatory conditions [1]. These radicals can deplete endogenous antioxidants, disrupt gene expression, and interfere with normal protein synthesis. It has been estimated that approximately 5% or more of inhaled oxygen (O_2_) is metabolized into reactive oxygen species (ROS), including superoxide anion (O_2_^•−^), hydrogen peroxide (H_2_O_2_), and hydroxyl radicals (OH^•^), predominantly through the mitochondrial electron transport chain. These ROS represent the major class of free radicals in living organisms [2].

Antioxidants play a critical role in neutralizing the deleterious effects of free radicals, thereby protecting the human body against infections and degenerative diseases [3]. They can be broadly categorized into two types: natural and synthetic. Common synthetic antioxidants include butylated hydroxyanisole (BHA), butylated hydroxytoluene (BHT) [4], tertiary butylhydroquinone (TBHQ) [5], and gallic acid esters [6, 7]. These compounds function by inhibiting oxidation reactions and can act as metal chelators, such as ethylenediaminetetraacetic acid (EDTA), to minimize metal-catalyzed oxidative damage [8]. However, concerns have been raised regarding the long-term safety of synthetic antioxidants, with some studies suggesting potential risks of mutagenesis and carcinogenesis [9]. As a result, there is growing scientific and consumer interest in identifying and utilizing naturally derived antioxidants as safer alternatives. These natural compounds offer promising potential in the prevention of oxidative stress-related diseases and are increasingly favored in the food, pharmaceutical, and nutraceutical industries [10].

ROS are critically involved in the pathophysiology of various human degenerative disorders, including aging, cancer, and neurodegenerative diseases such as Alzheimer's, Parkinson's, and Huntington's diseases [11]. Among these, hydrogen peroxide (H_2_O_2_) is a major ROS that contributes to cellular damage by promoting lipid peroxidation and inducing DNA strand breaks. In recent years, extensive research has been devoted to the discovery of naturally occurring bioactive compounds capable of mitigating oxidative stress and its associated cellular consequences [10]. Natural antioxidants exhibit a wide array of biochemical activities, including the inhibition of ROS generation, direct or indirect scavenging of free radicals, and modification of intracellular redox potential [12]. Antioxidants have been employed to impede apoptosis, initially perceived as being mediated by oxidative stress. Several antioxidant substances possess anticancer or anticarcinogenic properties [13]. Natural antioxidants derived from plants have attracted considerable scientific attention due to their broad therapeutic potential and their historical role in traditional medicine. Numerous phytochemicals have been shown to counteract oxidative stress and offer protection against a wide range of diseases. For instance, resveratrol, a polyphenolic compound found in grapes and other food sources, has demonstrated protective effects against oxidative damage and apoptosis and has been reported to inhibit carcinogenesis in murine models [14–16]. Similarly, epigallocatechin-3-gallate (EGCG), the principal catechin in green tea, has been shown to effectively scavenge free radicals and suppress the formation of carcinogen-induced tumors in rodent models of skin, lung, forestomach, and colon cancers [17–19]. Ginsenosides Rb1 and Rg3, isolated from *Panax ginseng*, have been reported to protect cultured rat cortical neurons from glutamate-induced neurotoxicity [20]. Furthermore, methanolic extracts of heat-processed neo-ginseng have been shown to reduce lipid peroxidation in rat brain homogenates triggered by ferric ions or ferric ions in combination with ascorbic acid. Another extensively studied phytochemical, curcumin, a yellow pigment derived from turmeric (*Curcuma longa* Linn., *Zingiberaceae*), possesses a diarylheptanoid structure and exhibits well-documented anticarcinogenic and antimutagenic effects in both *in vitro* systems and animal models [21].

Phenolic compounds derived from medicinal plants exhibit potent antioxidant activity and may shield cells against oxidative damage induced by free radicals. This class of antioxidant agents serves as free radical terminators and possesses scavenging abilities owing to their hydroxyl groups [22]. They are recognized as radical scavengers, metal chelators, reducing agents, hydrogen donors, and singlet oxygen quenchers. The interest in natural antioxidants has surged due to the toxicity associated with synthetic antioxidants such as BHA and BHT at relatively high doses, which restricts their therapeutic utility [23, 24].

*Cissampelos pareira* L. (Menispermaceae) is a climbing medicinal plant with a long history of ethnopharmacological use across Asia, Africa, and Latin America [25]. Traditionally known as the “midwife's herb” in Ayurveda, it has been employed to manage female reproductive health, gastrointestinal disorders, inflammatory diseases, and urinary conditions [26–29]. Scientific investigations have validated many of these uses, highlighting its wide spectrum of pharmacological activities. The plant exhibits strong anti-inflammatory and analgesic effects through the inhibition of inflammatory mediators, supported by bioactive alkaloids such as berberine and isoliensinine [25, 30, 31]. Its antimicrobial and antivenom properties have reinforced its role in treating infections and snakebites [32], while its cytotoxic and antimalarial activities offer promise for cancer and malaria therapy [33]. These findings support the ongoing exploration of *C. pareira* as a natural source of pharmacologically active compounds for potential use in phytotherapeutics and modern integrative medicine. Therefore, the present study aimed to evaluate the phytochemical content, antioxidant capacity, and anticancer activities of *C. pareira* L. stem and leaf extracts prepared using three different solvents—water, ethanol, and hexane—so as to explore the solvent-dependent variation in bioactivity and identify the most promising fractions for potential biomedical applications.

## 2. Materials and Methods

### 2.1. Materials

Folin & Ciocalteu's reagent, Trolox, gallic acid, ascorbic acid, 2,2′-azinobis (3-ethylbenzothiazoline 6-sulfonate) (ABTS), DPPH (2, 2-diphenyl-1-picrylhydrazyl), dimethyl sulfoxide (DMSO), sodium bicarbonate, and [3-(4,5-dimethylthiazol-2-yl)-2,5-diphenyl tetrazolium bromide] (MTT) were purchased from Sigma–Aldrich, Dulbecco's Modified Eagle Medium (DMEM) medium, fetal bovine serum (FBS), and an antibiotic mixture (penicillin–streptomycin) were procured from Gibco (Thermo Fisher Scientific, Waltham, MA, USA).

### 2.2. Plant Materials

Stems and leaves of *C. pareira* L. were gathered from forests in Chiang Kham district, Phayao Province, Thailand, in December 2024. A voucher specimen (No. 0023401) has been deposited in the herbarium of the Faculty of Pharmacy, Chiang Mai University, to serve as a reference for taxonomic verification.

### 2.3. Preparation of Plant Extracts

The dried stems and leaf powders of *C. pareira* L. were separately subjected to solvent extraction using distilled water, ethanol, and hexane. For each extraction, 10 g of plant material was macerated with 100 mL of the respective solvent (1:10 w/v) in a Schott bottle and left to stand at room temperature for 72 h with occasional shaking. The mixtures were then filtered through Whatman No. 1 filter paper. The resulting filtrates were concentrated under reduced pressure at 40°C using a rotary evaporator, frozen, and subsequently lyophilized to obtain dry extracts. The percentage yield was calculated based on the dry weight (DW) of the extract relative to the initial weight of the plant material. The extraction yield (%) was calculated using the following formula:

(1) $$\begin{array}{c} \text{\% Yield }=\;\left[\text{Dry weight of extract }\left(g\right)/\text{Dry weight of raw sample }\left(g\right)\right]\times 100. \end{array}$$

The stem extracts were labeled as CPSW (water), CPSH (hexane), and CPSE (ethanol), while the leaf extracts were labeled as CPLW (water), CPLH (hexane), and CPLE (ethanol), respectively.

### 2.4. Determination of Proximate Composition Analysis and Phytoconstituents

#### 2.4.1. Proximate Composition Analysis

The proximate composition of the samples, including moisture, ash, crude protein, crude fat, crude fiber, and carbohydrate contents, was determined following the official methods of the Association of Official Analytical Chemists (AOAC) [34]. All analyses were conducted in triplicate. Moisture content was determined by oven-drying the samples at 105°C until a constant weight was achieved. Ash content was measured by incinerating the samples in a muffle furnace at 550°C for 6 h. Crude protein content was analyzed using the Kjeldahl method. Crude fat was extracted using the Soxhlet extraction technique with petroleum ether (boiling point 40–60°C) as the solvent. After extraction, the solvent was evaporated by drying the flask in a hot air oven, followed by cooling and weighing to determine fat content. Crude fiber was analyzed by digesting defatted samples sequentially in dilute acid (0.25 N H_2_SO_4_ and 0.25 N NaOH) under reflux. The residue was rinsed successively with hot water, acetone, and 50% ethanol, dried at 130°C for 2 h, cooled, and weighed. Total carbohydrate content (%) was calculated by difference, using the following formula:

(2) $$\begin{array}{c} \text{\% Carbohydrate }=\;100-\left(\text{\% Moisture }+\text{ \% Fat }+\text{ \% Ash }+\text{ \% Crude fiber }+\text{ \% Protein}\right) \end{array}$$

The total energy value (kcal) was estimated based on the Atwater general factors, using the following equation:

(3) $$\begin{array}{c} \text{Energy content }\left(\text{Kcal}\right)=\left(4\times \text{\% Protein}\right)+\left(4\times \text{\% Carbohydrate}\right)+\left(9\times \text{\% Fat}\right) \end{array}$$

#### 2.4.2. Determination of Total Phenolic Contents (TPCs)

The TPC of the plant extracts was determined using the Folin–Ciocalteu colorimetric method. Briefly, 100 µL of each extract was mixed with 125 µL of Folin–Ciocalteu reagent, followed by the addition of 300 µL of 20% sodium carbonate (Na_2_CO_3_). The final volume was adjusted to 1 mL with double-distilled water and incubated at room temperature for 2 h. Absorbance was then measured at 760 nm using a UV-Vis spectrophotometer. Results were expressed as milligrams of gallic acid equivalents (mg GAE) per gram of DW of the extract.

#### 2.4.3. Determination of Total Flavonoid Contents (TFCs)

The TFC of the extracts was determined using the aluminum chloride colorimetric method. Briefly, 250 µL of the extract was mixed with 75 µL of 5% sodium nitrite (NaNO_2_) solution. After 6 min, 150 µL of 10% aluminum chloride (AlCl_3_) was added, followed by 500 µL of 1 M sodium hydroxide (NaOH) after a further 5 min. The final volume was adjusted to 2.5 mL with distilled water. Absorbance was measured at 510 nm using a UV-Vis spectrophotometer. The results were expressed as milligrams of catechin equivalents (mg CE) per gram of DW of the extract.

### 2.5. Antioxidant Ability Assays

#### 2.5.1. DPPH Radical Assay

The antioxidant activity of the extracts was assessed using the DPPH (2,2-diphenyl-1-picrylhydrazyl) radical scavenging assay, following established protocols [35]. A DPPH stock solution was prepared by dissolving 6.6 mg/mL of DPPH in ethanol and was stored in the dark until use. Serial dilutions of the test extracts, as well as standard antioxidants (ascorbic acid and Trolox), were prepared in ethanol at concentrations ranging from 0 to 20 mg/mL. For the assay, 180 μL of the DPPH solution and 20 μL of each test sample were dispensed into the wells of a 96-well microtiter plate, resulting in a final reaction volume of 200 μL. The plates were incubated at 37°C for 30 min to allow the reduction of the DPPH radical, indicated by a visible color change from deep violet to pale yellow. Absorbance was then measured at 540 nm using a microplate reader. Ethanol containing DPPH alone was used as the negative control, while ascorbic acid and Trolox served as positive standards. The radical scavenging activity was expressed as percentage inhibition and calculated using the following formula:

(4) $$\begin{array}{c} \text{\% Inhibition }=\;\left[\left(\text{Ac }-\text{ As}\right)\times 100\right]\;/\text{ Ac} \end{array}$$

where Ac represents the absorbance of the control and As represents the absorbance of the sample after the incubation period. Dose-response curves were generated by plotting the percentage inhibition against the concentrations of the test samples. The IC_50_ values, defined as the concentration required to inhibit 50% of DPPH radicals, were determined using nonlinear regression analysis in GraphPad Prism software.

#### 2.5.2. ABTS^•+^ Radical Assay

The antioxidant capacity of the extracts was evaluated using the ABTS^•+^ [2,2′-azinobis (3-ethylbenzothiazoline-6-sulfonic acid) diammonium salt] radical cation decolorization assay, with ascorbic acid and Trolox serving as reference antioxidants as previously published [35]. In this assay, 20 µL of extract, prepared in concentrations ranging from 0 to 20 mg/mL, was added to 2.0 mL of diluted ABTS^•+^ solution (adjusted to an initial absorbance of 0.700 ± 0.020 at 734 nm). The reaction mixture was incubated at room temperature for 5 min, and the absorbance was recorded at 734 nm using a UV-Vis spectrophotometer. A solvent blank containing all reagents except the test compound was used as the control. Each assay was performed in triplicate and repeated at least three times to ensure reproducibility. The percentage inhibition of ABTS^•+^ was calculated using the following equation:

(5) $$\begin{array}{c} \text{\% Inhibition }=\;\left[\left(\text{Ablank }-\text{ Asample}\right)\;/\text{ Ablank}\right]\times 100 \end{array}$$

where Ablank is the absorbance of the control reaction (containing all reagents except the test sample), and Asample is the absorbance in the presence of the extract. Dose-response curves were generated by plotting the percentage inhibition against the concentrations of the extracts. Ascorbic acid and Trolox were used as positive controls for comparative analysis. The radical scavenging activity and IC_50_ values were calculated in accordance with the procedure described for the DPPH assay.

### 2.6. Anticancer Activity

#### 2.6.1. Cell Culture

The human cell lines MCF-7 (estrogen receptor-positive breast cancer), MDA-MB-231 (triple-negative breast cancer), HEK293T (human embryonic kidney), and normal human dermal fibroblasts (NHDFs) were obtained from the American Type Culture Collection (ATCC, Manassas, VA, USA). All cell lines were cultured in DMEM, supplemented with 10% (*v*/*v*) FBS and 1× antibiotic solution to prevent microbial contamination. The cells were maintained under standard cell culture conditions at 37°C in a humidified incubator with 5% CO_2_. Media were replaced every 2–3 days, and cells were subcultured upon reaching 70%–80% confluency.

#### 2.6.2. MTT Assay

Cells were seeded into 96-well plates at a density of 5 × 10^3^ cells per well and allowed to adhere for 24 h under standard incubation conditions. Subsequently, the cells were treated with increasing concentrations (0–800 μg/mL) of *C. pareira* L. extracts and incubated for an additional 24 h. Following treatment, 20 μL of MTT reagent (3-(4,5-dimethylthiazol-2-yl)-2,5-diphenyl tetrazolium bromide) at a concentration of 5 mg/mL was added to each well, and the plates were incubated at 37°C for 2 h. After incubation, the supernatant was carefully removed, and 100 μL of DMSO was added to each well to dissolve the formazan crystals formed by viable cells. The contents were mixed thoroughly to ensure complete dissolution. Absorbance was measured at 570 nm using a microplate reader (Cytation 3, BioTek Instruments, Winooski, VT, USA). Cell viability was expressed as a percentage relative to untreated control cells, and the IC_50_ values of the extracts were calculated using nonlinear regression analysis with GraphPad Prism software (GraphPad Software, San Diego, CA, USA).

### 2.7. Statistical Analysis

All data are presented as mean ± standard deviation (SD) based on three independent experiments performed in triplicate. Statistical analysis was conducted using one-way analysis of variance (ANOVA), followed by Tukey's honestly significant difference (HSD) test for post hoc comparisons. A *p*-value <0.05 was considered statistically significant. All analyses were performed using GraphPad Prism software (version 9.0; GraphPad Software Inc., San Diego, CA, USA).

## 3. Results

### 3.1. Proximate Composition Analysis

The proximate composition of *C. pareira* L. stem and leaf is presented in Table 1. The leaf exhibited significantly higher values of moisture (5.64% ± 0.08%), crude protein (16.63% ± 0.10%), carbohydrate (53.93% ± 0.02%), and energy content (297.81 ± 3.80 kcal) compared to the stem (*p* < 0.05). In contrast, the stem had a significantly higher crude fiber content (48.02% ± 0.14%) than the leaf (14.33% ± 0.24%). The ash and crude fat contents showed no significant differences between the two plant parts. These differences suggest that the nutritional profile of *C. pareira* L. varies markedly between its stem and leaf, with the leaf providing higher protein and energy but the stem offering superior fiber content.

### 3.2. Solvent-Dependent Variation in Extraction Yield, TPC, and TFC of *C. pareira* L. Extracts

The extraction yields, TPC, and TFC of *C. pareira* L. stem and leaf extracts obtained using water, ethanol, and hexane are summarized in Table 2. Among the stem extracts, CPSE showed the highest yield (14.90%) and TPC (58.26 ± 2.56 mg GAE/g DW), while CPSW exhibited the highest TFC (27.23 ± 2.89 mg CE/g DW). CPSH showed the lowest values for both TPC (9.74 ± 3.84 mg GAE/g DW) and TFC (17.84 ± 6.03 mg CE/g DW), with a yield of only 0.42%. In the case of leaf extracts, CPLE yielded the highest TPC (95.73 ± 1.76 mg GAE/g DW) and TFC (82.68 ± 4.98 mg CE/g DW), with a yield of 11.70%. CPLW contained moderate flavonoid levels (40.04 ± 3.62 mg CE/g DW) but low phenolic content (12.04 ± 0.18 mg GAE/g DW), while CPLH showed the lowest TFC (8.25 ± 2.54 mg CE/g DW) and TPC (15.32 ± 1.07 mg GAE/g DW), with a yield of 1.17%. These results suggest that ethanol is the most effective solvent for extracting both phenolic and flavonoid compounds from *C. pareira* L., particularly from the leaves.

### 3.3. Antioxidant Activity of *C. pareira* L. Extracts Assessed by DPPH and ABTS Radical Scavenging Assays

The antioxidant activities of *C. pareira* L. extracts were evaluated using DPPH and ABTS^+^ radical scavenging assays, and the results are summarized in Table 3 as IC_50_ values. Lower IC_50_ values indicate higher antioxidant potential. Among all extracts, CPLE exhibited the most potent antioxidant activity, with IC_50_ values of 55.00 ± 1.93 µg/mL for DPPH and 25.77 ± 0.38 µg/mL for ABTS^+^. CPSE also demonstrated moderate antioxidant potential (IC_50_: 186.00 ± 15.44 µg/mL for DPPH; 48.72 ± 0.41 µg/mL for ABTS^+^). In contrast, CPSH and leaf (CPLH) showed negligible activity (IC_50_ > 400 µg/mL for both assays), similar to CPSW and CPLW in the DPPH assay. Trolox and ascorbic acid were used as positive controls, with IC_50_ values of 4.32 ± 0.32 and 4.57 ± 0.27 µg/mL for DPPH, and 4.42 ± 0.11 and 6.72 ± 0.15 µg/mL for ABTS^+^, respectively.

### 3.4. Anticancer Activity

**Figure 1:**
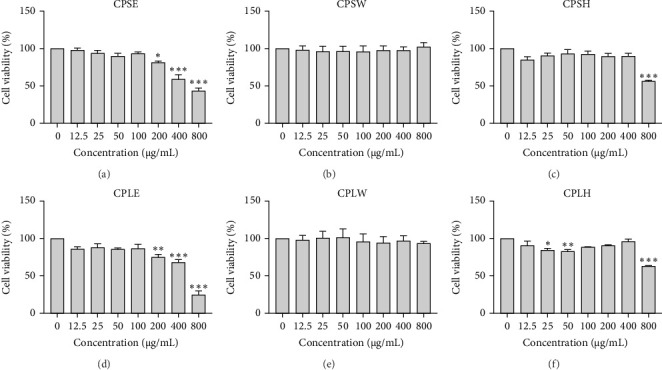
Effect of *Cissampelos. pareira* L. extracts on the viability of MCF-7 breast cancer cells. MCF-7 cells were treated with six different extracts of *C. pareira* L. at concentrations ranging from 0 to 800 µg/mL for 24 h: (A) stem ethanol extract (CPSE), (B) stem water extract (CPSW), (C) stem hexane extract (CPSH), (D) leaf ethanol extract (CPLE), (E) leaf water extract (CPLW), and (F) leaf hexane extract (CPLH). Cell viability was assessed using the MTT assay. Results are expressed as mean ± standard deviation (SD) from three independent experiments (*n* = 3). Statistical significance compared to untreated control is indicated as follows: *⁣*^*∗*^*p* < 0.05, *⁣*^*∗∗*^*p* < 0.01, *⁣*^*∗∗∗*^*p* < 0.001.

**Figure 2:**
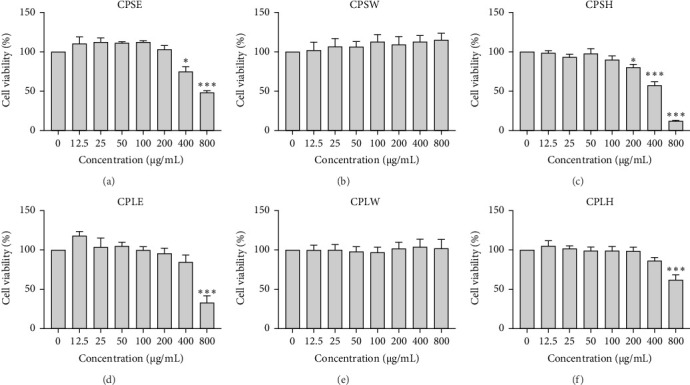
Effect of *Cissampelos. pareira* L. extracts on the viability of MDA-MB-231 triple-negative breast cancer cells. MDA-MB-231 cells were exposed to the same six extracts as described in Figure 1—CPSE (A), CPSW (B), CPSH (C), CPLE (D), CPLW (E), and CPLH (F)—at concentrations ranging from 0 to 800 µg/mL for 24 h. Cell viability was determined using the MTT assay. Data are presented as mean ± SD from three independent experiments (*n* = 3). Statistical significance compared to untreated control: *⁣*^*∗*^*p* < 0.05, *⁣*^*∗∗*^*p* < 0.01, *⁣*^*∗∗∗*^*p* < 0.001.

**Figure 3:**
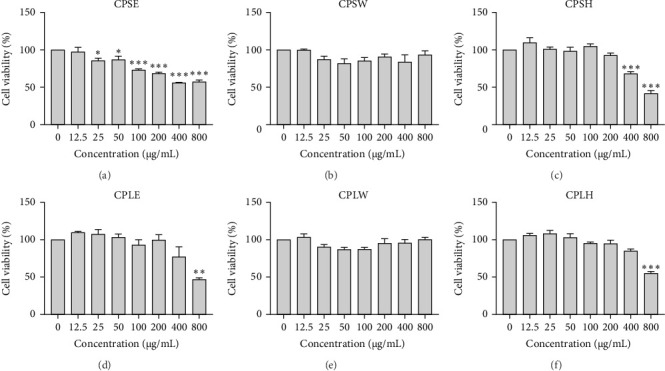
Effect of *Cissampelos. pareira* L. extracts on the viability of HEK293T human embryonic kidney cells. HEK293T cells were treated with CPSE (A), CPSW (B), CPSH (C), CPLE (D), CPLW (E), and CPLH (F) at concentrations ranging from 0 to 800 µg/mL for 24 h. The MTT assay was used to assess cell viability. Results are shown as mean ± SD from three independent experiments (*n* = 3). Statistical comparisons were made relative to the untreated control: *⁣*^*∗*^*p* < 0.05, *⁣*^*∗∗*^*p* < 0.01, *⁣*^*∗∗∗*^*p* < 0.001.

**Figure 4:**
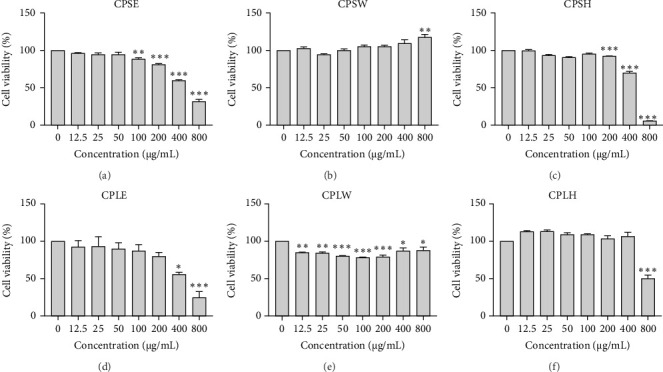
Effect of *Cissampelos. pareira* L. extracts on the viability of NHDF (normal human dermal fibroblast) cells. NHDF cells were exposed to CPSE (A), CPSW (B), CPSH (C), CPLE (D), CPLW (E), and CPLH (F) extracts at concentrations of 0 to 800 µg/mL for 24 h. Cell viability was evaluated using the MTT assay. Data are expressed as mean ± SD from three independent experiments (*n* = 3). Statistical significance compared to untreated control: *⁣*^*∗*^*p* < 0.05, *⁣*^*∗∗*^*p* < 0.01, *⁣*^*∗∗∗*^*p* < 0.001.

The cytotoxic effects of *C. pareira* L. extracts prepared using water, ethanol, and hexane were evaluated against human breast cancer cell lines (MCF-7 and MDA-MB-231) (Figures 1 and 2) and human embryonic kidney cells (HEK293T) (Figure 3) using the MTT assay. The IC_50_ values, representing the concentration required to inhibit 50% of cell viability after 24 h of exposure, are summarized in Table 4. Among all tested extracts, CPLE demonstrated the most potent cytotoxicity against MCF-7 cells with an IC_50_ of 310.9 ± 4.32 µg/mL, followed by CPSE with an IC_50_ of 472.2 ± 4.63 µg/mL. Similarly, for MDA-MB-231 cells, CPSE and CPLE showed moderate cytotoxicity with IC_50_ values of 385.4 ± 4.42 and 434.7 ± 4.57 µg/mL, respectively. CPSW and CPLW and CPSH and CPLH exhibited weak or negligible cytotoxic effects in all tested cancer cell lines, with IC_50_ values exceeding 800 µg/mL. Importantly, none of the extracts exhibited significant cytotoxicity against HEK293T normal cells within the tested concentration range (0–800 µg/mL) (Figure 4), indicating a level of selectivity toward cancerous cells. These results suggest that the ethanol extracts, particularly from the leaves, may contain bioactive compounds with selective anticancer properties, warranting further phytochemical and mechanistic investigations.

## 4. Discussion

The present study provides a comprehensive evaluation of the nutritional composition, phytochemical content, and bioactivity of *C. pareira* L. stem and leaf extracts. The results clearly indicate distinct differences between the two plant parts and highlight the influence of solvent polarity on extraction efficiency and biological activities. The leaves were found to be more nutrient-dense, with significantly higher protein, carbohydrate, and energy content than the stems, whereas the stems were exceptionally rich in crude fiber (around 48%, versus 14% in leaves). This nutritional partitioning is consistent with the physiological roles of leaves and stems; leaves often serve as sites of photosynthesis and metabolite storage (hence higher proteins and sugars), while stems provide structural support (hence higher fiber). Notably, both parts had comparable ash (mineral) and low-fat content, suggesting that *C. pareira* is generally a low-lipid plant. These compositional traits imply that the leaves could offer greater nutritional value, if used in diets or herbal formulations, whereas the high fiber in stems might be useful for digestive health, but could also dilute the extractable phytochemicals. The choice of solvent had a profound impact on extraction yield and phytochemical recovery. Ethanol proved to be the most effective solvent overall, yielding the highest extractable matter and phenolic content from both stems and leaves. For instance, CPSE yielded 14.9% extract with a TPC of ~58 mg GAE/g DW, and CPLE yielded 11.7% with a remarkably high phenolic content of ~96 mg GAE/g DW. In contrast, water extracts yielded moderately less (around 9%–12% with much lower phenolics, especially in leaves), and hexane (nonpolar) extracts gave minimal yields (<2%) with negligible phenolic recovery. These findings are in agreement with the general understanding that moderately polar organic solvents (e.g., ethanol or hydroalcoholic mixtures) are most efficient for extracting polyphenols from plant matrices [36, 37]. Polar solvents penetrate the plant tissue and solubilize phenolics and flavonoids more effectively than water alone or nonpolar solvents [38]. Interestingly, the TFC did not always parallel the phenolics; in stem extracts, water pulled out slightly higher flavonoid levels than ethanol (27.2 vs. 19.9 mg CE/g DW), although this difference was not statistically significant. This suggests that certain flavonoids in the stem might be more water-soluble (perhaps as glycosides), whereas in the leaves, ethanol was vastly superior, yielding ~82.7 mg CE/g DW (over twice that of water extracts). The hexane extracts of both stems and leaves had the lowest TPC and TFC, indicating that most bioactive phenolics and flavonoids in *C. pareira* are polar or mid-polar compounds, not readily extracted by nonpolar solvents [39, 40]. This solvent-dependent phytochemical profile aligns with other reports on *C. pareira*, where hydroalcoholic extracts were found to contain abundant alkaloids and flavonoids [41–44]. Corresponding to the phytochemical content, the antioxidant activity of the extracts was strongly solvent- and organ-dependent. CPLE, which contained the highest phenolic and flavonoid levels, exhibited the most potent free radical scavenging capacity (IC_50_ ~55 µg/mL for DPPH and ~26 µg/mL for ABTS^+^)—markedly better than any other extract. The CPSE showed moderate activity (DPPH IC_50_ ~186 µg/mL), while water and hexane extracts from both parts were weak antioxidants (DPPH IC_50_ > 400 µg/mL). These results underscore the positive correlation between phenolic content and antioxidant potency, as widely documented in plant phytochemistry. One curious observation was that the leaf water extract, despite its low phenolic content, showed a relatively strong ABTS^+^ radical scavenging (IC_50_ ~56 µg/mL), while remaining inactive against DPPH radicals. This discrepancy can be explained by the differences between the two antioxidant assays. The ABTS^+^ radical cation is soluble in both aqueous and organic media and can be scavenged by a wide range of antioxidants, including hydrophilic compounds, whereas the DPPH radical is typically measured in organic solvents and is more accessible to lipophilic antioxidants [45, 46]. The leaf water extract likely contains highly polar antioxidants (e.g., ascorbic acid, phenolic glycosides, or other hydrophilic metabolites) that effectively quench ABTS^+^, but either cannot interact with DPPH in a hydrophobic medium or react too slowly to register a low IC_50_ [47]. This highlights the importance of using multiple assays to gauge antioxidant activity. Overall, our findings indicate that *C. pareira* leaves, especially when extracted in ethanol, are a rich source of natural antioxidants capable of neutralizing free radicals. This corroborates the plant's traditional use in treating inflammation and wounds [48, 49], conditions where oxidative stress is often a contributing factor.

*In vitro* cytotoxicity assays against two human breast cancer cell lines (MCF-7 and MDA-MB-231) revealed that *C. pareira* harbors constituents with mild to moderate anticancer activity, particularly in the ethanol extracts. The ethanol leaf extract was the most active, inhibiting MCF-7 cell proliferation with an IC_50_ of ~311 µg/mL, followed by the ethanol stem extract (IC_50_ ~472 µg/mL). Activity against the more aggressive MDA-MB-231 cells was slightly weaker (IC_50_ ~435 µg/mL) for leaf ethanol, ~385 µg/mL for stem ethanol. In contrast, the aqueous and hexane extracts showed little to no cytotoxic effect on these cancer cells (IC_50_ > 800 µg/mL). At first glance, the hexane stem extract appeared to have some cytotoxic potency in the summary of results, but closer scrutiny shows it lacked specificity—it was equally or even more toxic to noncancerous cells. Indeed, none of the extracts exhibited significant toxicity toward an immortalized human kidney cell line (HEK293T) up to the maximum tested concentration (800 µg/mL), except the hexane stem extract, which reduced HEK293T viability in the same concentration range (IC_50_ ≈ 311 µg/mL), comparable to its effect on MCF-7). Moreover, the ethanol leaf extract, while active against MCF-7, showed an IC_50_ of ~434 µg/mL in NHDFs, a higher value indicating lower toxicity than to the breast cancer cells. Together, these data suggest a degree of selectivity of the ethanol extracts for cancer cells over normal cells, whereas the hexane stem extract contains nonspecific cytotoxins. Selectivity is a crucial attribute for potential anticancer agents; it is preferable for a compound to target cancer cells while sparing normal healthy cells. The relatively selective action of the ethanol extracts (particularly CPLE) is encouraging and may be due to the presence of certain phenolic or alkaloid compounds that induce apoptosis or inhibit proliferation preferentially in cancer cells. This phenomenon has been observed with other plant-derived molecules; for example, the green tea polyphenol EGCG and related antioxidants can induce apoptosis more readily in cancer cells than in normal cells, possibly because cancer cells exhibit higher baseline oxidative stress and are more susceptible to further redox imbalance. It is noteworthy that the potency of *C. pareira* extracts in this study is moderate—IC_50_ in the few-hundred µg/mL range—which is not unusual for crude plant extracts. By comparison, a methanolic extract of *C. pareira* whole plant was reported to have an IC_50_ of ~95 µg/mL against Dalton's lymphoma cells *in vitro* and to significantly prolong the survival of tumor-bearing mice *in vivo* [41]. That study attributed the antitumor effect at least partly to antioxidant mechanisms and the ability of the extract to modulate oxidative stress in the host. The lower IC_50_ in the Dalton's lymphoma model may be due to differences in extraction solvent (methanol can extract a wide range of bioactives) or inherent sensitivity of the cell line. Regardless, our findings and those of prior studies collectively demonstrate that *C. pareira* possesses genuine anticancer potential, meriting further investigation.

The differential cytotoxic profiles between the leaf and stem extracts of *C. pareira* hint at differences in their chemical constituents. The superior activity of the leaf ethanol extract against MCF-7 aligns with its higher content of flavonoids and phenolics, many of which are known to exert antiproliferative effects through antioxidant-dependent and independent pathways [50]. Flavonoids such as quercetin, kaempferol, or plant-derived lignans can arrest the cell cycle and induce apoptosis in breast cancer cells, as shown in various studies, and could be present in the leaf extract. Additionally, *C. pareira* is rich in isoquinoline alkaloids characteristic of the Menispermaceae family [51]. Notably, compounds like cissampareine (also known as cissampeline) and pareirubrines A and B have been isolated from *C. pareira* and related species. Cissampareine, a bisbenzylisoquinoline alkaloid, has demonstrated significant cytotoxic activity against nasopharyngeal carcinoma cells *in vitro* [52]. Similarly, pareirubrine A—a novel tropoloisoquinoline alkaloid from *C. pareira*—was reported to exhibit potent antileukemic activity [53]. These alkaloids, being relatively nonpolar, may concentrate more in ethanol or even hexane extracts of roots and stems. It is plausible that the hexane stem extract's cytotoxicity is due to such alkaloidal constituents or other hydrophobic toxins (e.g., certain terpenoids or sterols). However, the lack of selectivity of that fraction, as evidenced by equal toxicity to normal cells, raises concerns, and suggests it contains general cytotoxic agents (perhaps membrane-disrupting compounds) rather than targeted anticancer molecules. In contrast, the ethanol extracts likely contain a complex mixture of polyphenols and alkaloids that together produce a cytotoxic effect more restricted to cancer cells, possibly through mechanisms like inducing oxidative stress selectively in cancer cells, triggering apoptosis pathways, or modulating signaling networks in a way that normal cells can better resist. Further phytochemical investigation is needed to isolate and identify the bioactive molecules in these extracts and to elucidate their mechanisms of action.

## 5. Conclusions

In summary, our study establishes that *C. pareira* L. leaves and stems possess distinct yet valuable phytochemical profiles. Ethanol extraction maximizes the yield of phenolics and flavonoids, translating into strong antioxidant activity and a measurable, selective cytotoxic effect against breast cancer cells *in vitro*. These properties validate some of the plant's traditional medicinal claims and open up possibilities for its incorporation into modern health products. Conversely, nonpolar extracts, while containing potent cytotoxins, lack specificity and underscore the importance of directed fractionation in the search for safe therapeutic agents. Taken together, the evidence positions *C. pareira* as a potential source of natural antioxidant nutraceuticals and provides a rationale for further development of its bioactive constituents—particularly the flavonoid-rich leaf extracts and alkaloid-rich fractions—as leads in cancer prevention or adjunct therapy, pending more detailed pharmacological investigations.

## Figures and Tables

**Table 1:** Proximate composition of the stem and leaf of *Cissampelos. pareira* L.

Constituents (%)	Stem	Leaf
Moisture	4.54 ± 0.13^a^	5.64 ± 0.08^b^
Ash	5.65 ± 0.15^b^	7.74 ± 0.10^b^
Crude protein	8.75 ± 0.17^b^	16.63 ± 0.10^f^
Crude fat	0.69 ± 0.11^a^	1.73 ± 0.22^a^
Carbohydrate	32.31 ± 0.47^c^	53.93 ± 0.02^g^
Crude fiber	48.02 ± 0.14^d^	14.33 ± 0.24^f^
Energy value (Kcal)	170.57 ± 2.07^e^	297.81 ± 3.80^h^

*Note*: Values are expressed as mean ± standard deviation (SD) based on triplicate analyses. Different superscript letters within the same row and column indicate statistically significant differences between stem and leaf (*p* < 0.05), as determined by two-way ANOVA followed by Tukey's HSD test.

**Table 2:** Extraction yield, total phenolic content (TPC), and total flavonoid content (TFC) of stem and leaf extracts of *Cissampelos. pareira* L. using different solvents.

Extracts	Yield (%)	Total phenolic content (mg GAE/g DW)	Total Flavonoids content (mg CE/g DW)
CPSE	14.90	58.26 ± 2.56^a^	19.92 ± 2.63^a^
CPSW	9.40	37.38 ± 0.99^b^	27.23 ± 2.89^a^
CPSH	0.42	9.74 ± 3.84^c^	17.84 ± 6.03^a^
CPLE	11.70	95.73 ± 1.76^d^	82.68 ± 4.98^b^
CPLW	11.50	12.04 ± 0.18^c^	40.04 ± 3.62^c^
CPLH	1.17	15.32 ± 1.07^c^	8.25 ± 2.54^d^

*Note:* Values are expressed as mean ± standard deviation (SD) based on triplicate determinations. TPC is reported in mg gallic acid equivalent (GAE) per gram of dry extract, and TFC is reported in mg catechin equivalent (CE) per gram of dry extract. Different superscript letters within the same column indicate statistically significant differences (*p* < 0.05) based on one-way ANOVA followed by Tukey's HSD test.

Abbreviations: CPLE, leaf extract in ethanol; CPLH, leaf extract in hexane; CPLW, leaf extract in water; CPSE, stem extract in ethanol; CPSH, stem extract in hexane; CPSW, *C. pareira* stem extract in water.

**Table 3:** Antioxidant activities of *Cissampelos. pareira* L. extracts evaluated by DPPH and ABTS radical scavenging assays.

Extracts	IC_50_ concentration (μg/mL)	
	DPPH	ABTS^+^
CPSE	186.00 ± 15.44	48.72 ± 0.41
CPSW	>400	191.27 ± 13.87
CPSH	>400	>400
CPLE	55.00 ± 1.93	25.77 ± 0.38
CPLW	>400	56.10 ± 1.36
CPLH	>400	>400
Trolox	4.32 ± 0.32	4.42 ± 0.11
Ascorbic acid	4.57 ± 0.27	6.72 ± 0.15

*Note*: Values are expressed as mean ± standard deviation (SD), based on three independent experiments (*n* = 3). IC_5_₀ represents the concentration of extract required to scavenge 50% of DPPH or ABTS^+^ radicals. A lower IC_5_₀ value indicates stronger antioxidant activity. Trolox and ascorbic acid were used as standard antioxidants.

Abbreviations: CPLE, leaf extract in ethanol; CPLH, leaf extract in hexane; CPLW, leaf extract in water; CPSE, stem extract in ethanol; CPSH, stem extract in hexane; CPSW, *C. pareira* stem extract in water.

**Table 4:** Cytotoxic activities (IC_5_₀, µg/mL) of water, ethanol, and hexane extracts of *Cissampelos. pareira* L. against MCF-7, MDA-MB-231, and HEK293T cell lines evaluated by MTT assay.

Extracts	IC_50_ (µg/mL)			
	MCF-7	MDA-MB-231	NHDF	HEK-293T
CPSE	618.40 ± 4.83	748.00 ± 4.98	501.10 ± 4.67	>800
CPSW	>800	>800	>800	>800
CPSH	472.2 ± 4.63	385.4 ± 4.42	446.30 ± 4.60	310.9 ± 4.32
CPLE	488.60 ± 4.66	650.60 ±	434.70 ± 4.57	746.80 ± 3.11
CPLW	>800	>800	>800	>800
CPLH	>800	>800	>800	>800

*Note*: Values are expressed as mean ± standard deviation (SD) from three independent experiments (*n* = 3). IC_5_₀ represents the concentration of extract required to inhibit 50% of cell viability, as determined by the MTT assay after 24 h of treatment. Lower IC_5_₀ values indicate higher cytotoxic activity.

Abbreviations: CPLE, leaf extract in ethanol; CPLH, leaf extract in hexane; CPLW, leaf extract in water; CPSE, stem extract in ethanol; CPSH, stem extract in hexane; CPSW, *C. pareira* stem extract in water.

## Data Availability

The data that support the findings of this study are available upon request from the corresponding author.
